# Graph prolongation convolutional networks: explicitly multiscale machine learning on graphs with applications to modeling of cytoskeleton

**DOI:** 10.1088/2632-2153/abb6d2

**Published:** 2020-12-01

**Authors:** Cory B Scott, Eric Mjolsness

**Affiliations:** Department of Computer Science, University of California Irvine, Irvine, California, United States of America

**Keywords:** machine learning, graph convolutional networks, molecular dynamics, microtubules

## Abstract

We define a novel type of ensemble graph convolutional network (GCN) model. Using optimized linear projection operators to map between spatial scales of graph, this ensemble model learns to aggregate information from each scale for its final prediction. We calculate these linear projection operators as the infima of an objective function relating the structure matrices used for each GCN. Equipped with these projections, our model (a Graph Prolongation-Convolutional Network) outperforms other GCN ensemble models at predicting the potential energy of monomer subunits in a coarse-grained mechanochemical simulation of microtubule bending. We demonstrate these performance gains by measuring an estimate of the Floating Point OPerations spent to train each model, as well as wall-clock time. Because our model learns at multiple scales, it is possible to train at each scale according to a predetermined schedule of coarse vs. fine training. We examine several such schedules adapted from the algebraic multigrid literature, and quantify the computational benefit of each. We also compare this model to another model which features an optimized coarsening of the input graph. Finally, we derive backpropagation rules for the input of our network model with respect to its output, and discuss how our method may be extended to very large graphs.

## 1. Introduction

### 1.1. Convolution and graph convolution

Recent successes of deep learning have demonstrated that the inductive bias of convolutional neural networks (CNNs) makes them extremely efficient for analyzing data with an inherent grid structure, such as images or video. In particular, many applications use these models to make per-node (per-pixel) predictions over grid graphs: examples include image segmentation, optical flow prediction, anticipating motion of objects in a scene, and facial detection/identification. Further work applies these methods to emulate physical models, by discretizing the input domain. Computational Fluid Dynamics and other scientific tasks featuring partial differential equations (PDEs) or ordinary differential equations (ODEs) on a domain discretized by a rectangular lattice have seen recent breakthroughs applying machine learning models, like CNNs to handle data which is structured this way. These models learn a set of local filters whose size is much smaller than the size of the domain—these filters may then be applied simultaneously across the entire domain, leveraging the fact that at a given scale the local behavior of the neighborhood around a pixel (voxel) is likely to be similar at all grid points.

Graph convolutional networks (GCNs) are a natural extension of the above idea of image ‘filters’ to arbitrary graphs rather than nD grids, which may be more suitable in some scientific contexts. Intuitively, GCNs replace the image filtering operation of CNNs with repeated passes of: (1) aggregation of information between nodes according to some structure matrix (2) non-linear processing of data at each node according to some rule (most commonly a flat neural network which takes as separate input(s) the current vector at each node). We refer the reader to a recent survey by Bacciu et al (2019) for a more complete exploration of the taxonomy graph neural networks.

### 1.2. Microtubules

**Figure 1.**
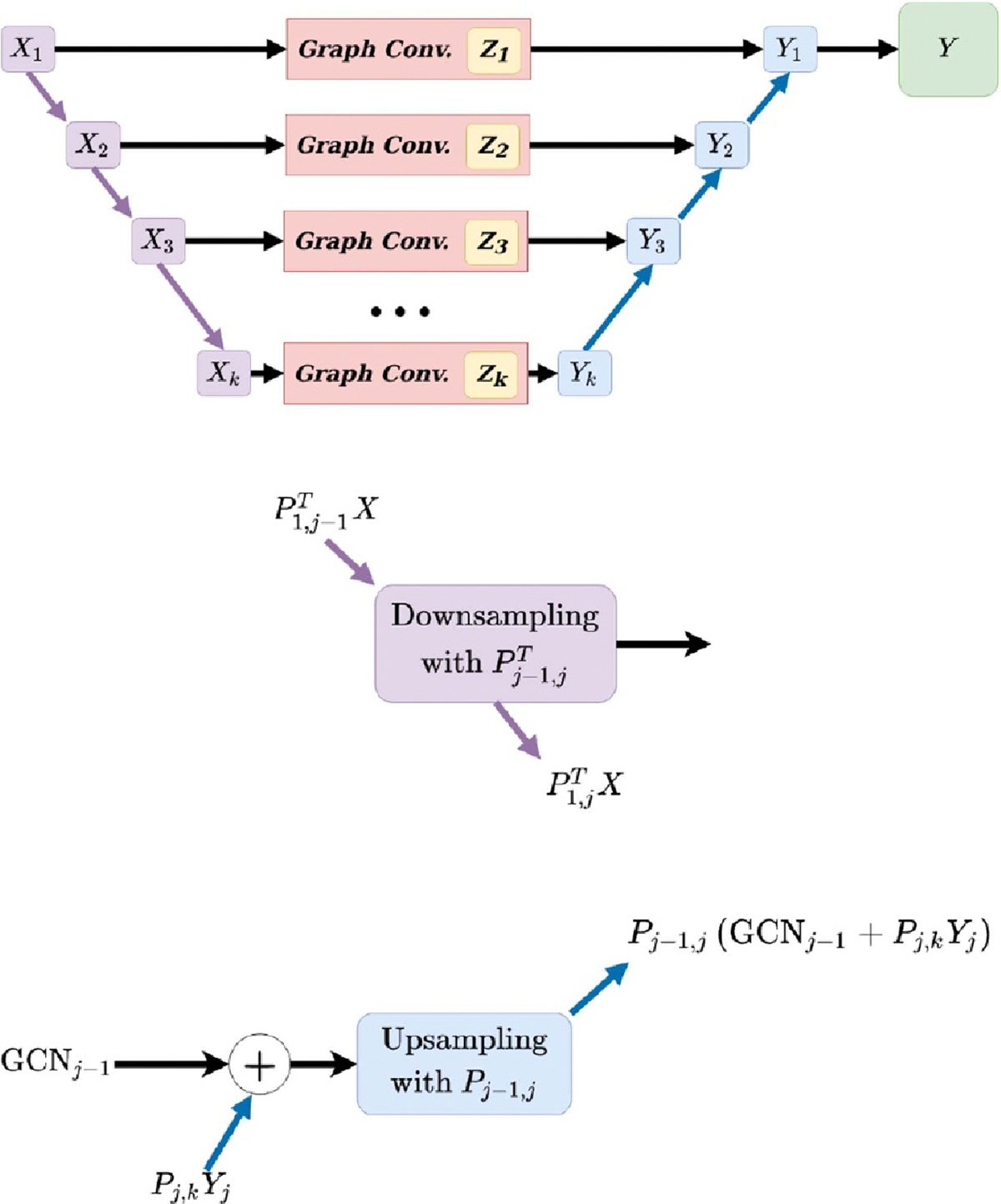
Top: Schematic of GPCN model. Data matrix X is fed into the model and repeatedly coarsened using optimized projection matrices $P_{ik}$, illustrated by purple arrows. These coarsened data matrices are separately fed into GCN models, producing predictions at each scale. Each blue arrow represents an upsample-and-add operation, where the upsampling is performed with the transpose of the $P_{ik}$. The final output of the ensemble is the projected sum of the outputs of each component GCN. Middle and bottom: mathematical details of upsampling and downsampling steps from top diagram. See equation (2) for details.

As an example of a dataset whose underlying graph is not a grid, we consider a coarse-grained simulation of a microtubule. Microtubules (MTs) are self-assembling nanostructures, ubiquitous in living cells, that along with actin filaments comprise a major portion of the dynamic cytoskeleton governing cell shape and mechanics. Whole-MT biomechanical models would be a useful tool for modeling cytoskeletal dynamics at the cellular scale. MTs play important structural roles during cell division, cell growth, and separation of chromosomes (in eukaryotic cells) (Chakrabortty et al 2018). MTs are comprised of a lattice structure of two conformations (α and β) of tubulin. Free-floating tubulin monomers associate energetically into dimer subunits, which then associate head-to-tail to form long chain-like complexes called *protofilaments*. Protofilaments associate side-to side in a sheet; at some critical number of protofilaments (which varies between species and cell type) the sheet wraps closed to form a repeating helical lattice with a seam. See (Pampaloni and Florin 2008, Page 303, figure 1). Key properties of MTs are:

#### Dynamic instability:

MTs grow from one end by attracting free-floating tubulin monomers (VanBuren et al 2005). MTs can spontaneously enter a ‘catastrophe’ phase, in which they rapidly unravel, but can also ‘rescue’ themselves from the catastrophe state and resume growth (Gardner et al 2013, Shaw et al 2003).

#### Interactions:

MTs interact with one another: they can dynamically avoid one another during the growth phase, or collide and bundle up, or collide and enter catastrophe (Tindemans et al 2014). The exact mechanism governing these interactions is an area of current research.

#### Structural strength:

MTs are very stiff, with a Young’s Modulus estimated at ≈1 GPa for some cases (Pampaloni and Florin 2008). This stiffness is thought to play a role in reinforcing cell walls (Kis et al 2002).

In this work we introduce a model which learns to reproduce the dynamics of a graph signal (defined as an association of each node in the network with a vector of discrete or real-valued labels) at multiple scales of graph resolution. We apply this model framework to predict the potential energy of each tubulin monomer in a simplified mechanochemical simulation of a microtubule. This trial dataset illustrates the efficiency of our proposed type of GCN and is a solid proof-of-concept for applying this model to more biologically accurate microtubule models in the future. In the next section, we discuss the wide variety of MT simulations which have been previously studied.

### 1.3. Simulation of MTs and prior work

Non-continuum, non-event-based simulation of large molecules is typically done by representing some molecular subunit as a particle/rigid body, and then defining rules for how these subunits interact energetically. Molecular dynamics (MD) simulation is an expansive area of study and a detailed overview is beyond the scope of this paper. Instead, we describe in general terms some basic ideas relevant to the numerical simulation detailed in section 3.1. Simulation of MTs is an area of active research, and there are many fundamental questions yet to be answered. A brief review of previous MT simulation studies (Stewman and Ma 2018, Gardner et al 2008, Molodtsov et al 2005, VanBuren et al 2005, Wang and Nogales 2005, Margolin et al 2012) finds a wide variety of different simulation techniques and assumptions. For this reason, we choose a simple model which is in a qualitative sense the ‘lowest common denominator’ of many of these models. Our MT simulation is a fixed structure of tubulin with energy terms defined only for tubulin-tubulin associations (consisting of angle and edge length constraints between monomers). We simulated the behavior of this structure under bending load in the MD software package LAMMPS (Plimpton 1993) using Verlet integration (Verlet 1967) and an implicit surrounding solvent (Schneider and Stoll 1978). For more details of our simulation, see section 3.1 and the source code, available in the Supplementary Material accompanying this paper (available online at stacks.iop.org/MLST/1/015001/mmedia). Each timestep of our simulator produces a vector consisting of each monomer’s contribution to the total potential energy of the structure at that timestep, as detailed in section 3.1. This vector is the target output we want our machine learning model to predict. In this work, we apply GCNs, trained via a method we introduce, to predict these energy values for a section of microtubule.

## 2. Model architecture and mathematical details

### 2.1. Model description

Many approaches to scientific problems benefit from the use of *multiscale* analysis: separating the behavior at hand into multiple scale lengths and analyzing each separately. We expect in general to have different phenomena at different scales, therefore necessitating varying treatments; a typical example would be a hybrid computational mechanics solver which uses both a continuum model at the largest spatial scale, but models spatially smaller interactions with an all-atom simulation (Stüben 2001, Wesseling and Oosterlee 2001). Even when phenomena are the same across multiple spatial scales (i.e. solving the Navier–Stokes equations on irregular domains (Raw 1996)) we expect to see acceleration of simulations when we use a multiscale architecture, as in the case of Multigrid solvers for iterative systems. These methods work on the premise that it if the wavelength of an error is large in comparison to the scale length considered by a solver, it may take many iterative steps at that scale to resolve the error. It is therefore advantageous to resolve errors at a scale similar to their characteristic wavelength, which is accomplished by building a hierarchy of solvers which each address error at a particular scale length. The exact method for reduction in error (a ‘smoothing’ step) is problem dependent; however, strategies for stepping between spatial scales have been invented, with good theoretical guarantees for accelerated error reduction of the entire system.

It is here necessary to note that the scheduling dictates which scale of error is reduced at a given step in the algorithm. In multigrid methods, the actual fine-to-coarse mapping (or vice versa) is given by multiplying the current solution by either a restriction or prolongation matrix, respectively. Typically these matrices are constrained, for example to be norm-preserving. This is similar in both motivation and practice to the matrix multiplication we use in our model architecture, detailed below and in section 2.4.

Multiscale architectures are also a staple of machine learning methods. CNNs, as described in section 1.1, are an example of such a system: features are propagated through the network so that the nodes in the final layer are aggregating information from a wide visual area. Motivated by both CNNs and the multiscale method literature, we develop a model which uses a multiscale architecture to learn MD at multiple spatial scales. Input is coarsened to each of these scales by applying an optimized linear projection (for details of this optimization, see section 3.2). At each scale, a GCN processes that scale’s information, analogous to the lateral connections in U-Net (Ronneberger et al 2015). Again analogously to the ‘upscaling’ connection in U-Net, the output of these GCNs is upsampled using the inverse of the same optimized linear projection used in the prior downsampling step. These outputs are all summed to produce a final model prediction at the finest scale. In the rest of this section, we first provide some general mathematical background (section 2.2), formally define Graph Convolution (section 2.3), and finally use these definitions to formally specify our model architecture in (section 2.4).

### 2.2. Mathematical background

#### Definitions:

For all basic terms (graph, edge, vertex, degree) we use standard definitions. We use the notation ${\left\{x_{i}\right\}}_{i=a}^{b}$ to represent the sequence of $x_{i}$ indexed by the integers a,a+1,a+2,…b. When X is a matrix, we will write ${\left[X\right]}_{ij}$ to denote the entry in the ith row, jth column.

#### Graph Laplacian:

The graph Laplacian is the matrix given by L(G)=A(G)-diag(A(G)⋅1) where A(G) is the adjacency matrix of G, and 1 is an appropriately sized vector of 1 s. The graph Laplacian is given by some authors as the opposite sign.

#### Linear graph diffusion distance (GDD):

Given two graphs $G_{1}$ and $G_{2}$, with $\left|G_{1}\right|\leq \left|G_{2}\right|$ the Linear GDD $D\left(G_{1},G_{2}\right)$ is given by:

(1) $$D\left(G_{1},G_{2}\right)=\underset{\begin{array}{c} P|𝒞(P)\\ \alpha >0 \end{array}}{inf}{\left\|\frac{1}{\alpha }PL\left(G_{1}\right)-\alpha L\left(G_{2}\right)P\right\|}_{F}$$

where 𝒞(P) represents some set of constraints on P,α is a scalar with α>0, and $\|\cdot {\|}_{F}$ represents the Frobenius norm. We take 𝒞(P) to be orthogonality: $P^{T}P=I$. Note that since in general P is a rectangular matrix, it may not be the case that $PP^{T}=I$. Unless stated otherwise all P matrices detailed in this work were calculated with α=1, using the procedure laid out in the following section, in which we briefly detail an algorithm for efficiently computing the distance in the case where α is allowed to vary. The efficiency of this algorithm is necessary to enable the computation of the LGDD between very large graphs, as discussed in section 5.3.

#### Prolongation matrix:

we use the term ‘prolongation matrix’ to refer to a matrix which is the optimum of the minimization given in the definition of the LGDD.

### 2.3. Graph convolutional layer definition

We follow the GCN formulation given by Kipf and Welling (2016). Assuming an input tensor X of dimensions n×F (where n is the number of nodes in the graph and F is the dimension of the label at each node), we inductively define the layerwise update rules for a GCN $GCN\left(Z_{i},X,{\left\{\theta_{l}^{\left(i\right)}\right\}}_{l=1}^{m}\right)$ as:

$$\begin{array}{l} X_{0}=X\\ X_{m}=g_{m}\left(Z_{i}X_{m-1}W_{m}^{\left(i\right)}+b_{m}^{\left(i\right)}\right), \end{array}$$

where $g_{m}$ is the activation function of the mth layer.

### 2.4. Graph prolongation convolutional networks

The model we propose is an ensemble of GCNs at multiple scales, with optimized projection matrices performing the mapping in between scales (i.e. between ensemble members). More formally, Let ${\left\{G_{i}\right\}}_{i=1}^{k}$ represent a sequence of graphs with $\left|G_{1}\right|\geq \left|G_{2}\right|\ldots \geq \left|G_{k}\right|$, and let ${\left\{Z_{i}=z\left(G_{i}\right)\right\}}_{i=1}^{k}$ be their structure matrices (for some chosen method z of calculating the structure matrix given the graph). In all experiments in this paper, we take z(G)=L(G), the graph Laplacian, as previously defined^1^. In an ensemble of GCNs, let $\theta_{l}^{\left(i\right)}=\left\{W_{l}^{\left(i\right)},b_{l}^{\left(i\right)}\right\}$ represent the parameters (filter matrix and bias vector) in layer l of the ith network.

When i=j-1, let $P_{i,j}$ be an optimal (in either the sense of GDD, or in the sense we detail in section 4.5) prolongation matrix from $L\left(G_{j}\right)$ to $L\left(G_{i}\right)$, i.e. $P_{i,j}=arg{inf}_{P|𝒞(P)}||PL\left(G_{j}\right)-L\left(G_{i}\right)P{\|}_{F}$. Then, for i<j-1, let $P_{i,j}$ be shorthand for the matrix product $P_{i,i+1}P_{i+1,i+2}\ldots P_{j-1,j}$. For example, $P_{1,4}=P_{1,2}P_{2,3}P_{3,4}$.

Our multiscale ensemble model is then constructed as:

(2) $$GPCN\left({\left\{Z_{i}\right\}}_{i=1}^{k},X,{\left\{{\left\{\theta_{l}^{\left(i\right)}\right\}}_{l=1}^{m_{i}}\right\}}_{i=1}^{k},{\left\{P_{i,i+1}\right\}}_{i=1}^{k-1}\right)\;\;=GCN\left(Z_{1},X,{\left\{\theta_{l}^{\left(1\right)}\right\}}_{l=1}^{m_{1}}\right)\;\;+\sum_{i=2}^{k}P_{1i}GCN\left(Z_{i},P_{1i}^{T}X,{\left\{\theta_{l}^{\left(i\right)}\right\}}_{l=1}^{m_{i}}\right)$$

This model architecture is illustrated in figure 1. When the P matrices are constant/fixed, we will refer to this model as a GPCN, for Graph Prolongation-Convolutional Network. However, we find in our experiments in section 4.5 that validation error is further reduced when the P operators are tuned during the same gradient update step which updates the filter weights, which we refer to as an ‘adaptive’ GPCN or A-GPCN. We explain our method for choosing $Z_{i}$ and optimizing P matrices in section 4.5.

## 3. Dataset generation and reduced model construction

In this section we describe some of the ancillary numerical results needed to reproduce and understand our main machine learning results in section 4.

### 3.1. Dataset

In this section we detail the process for generating the simulated microtubule data for comparison of our model with other GCN ensemble models. Our MT structure has 13 protofilaments (each 48 tubulin monomers long). As in a biological microtubule, each tubulin monomer is offset (along the axis parallel to the protofilaments) from its neighbors in adjacent protofilaments, resulting in a helical structrure with a pitch of 3 tubulin units. We refer to this pitch as the ‘offset’ in section 3.3. Each monomer subunit (624 total) is represented as a point mass of 50 Dalton (8.30×10^−15^ ng). The diameter of the whole structure is 26 nm, and the length is ≈260 nm. The model itself was constructed using Moltemplate (Jewett et al 2013), a tool for constructing large regular molecules to be used in LAMMPS simulations. Our Moltemplate structure files were organized hierarchically, with: tubulin monomers arranged into α-β dimer pairs; which were then arranged into rings of 13 dimers; which were then stacked to create a molecule 48 dimers long. Note that this organization has no effect on the final LAMMPS simulation: we report it here for reproducibility, as well as providing the template files in the supplementary material accompanying this paper.

For this model, we define energetic interactions for angles and associations only. No steric or dihedral interactions were used: for dihedrals, this was because the lattice structure of the tube meant any set of four molecules contributed to multiple, contradictory dihedral interactions^2^. Interaction energy of an association b was calculated using the ‘harmonic’ bond style in LAMMPS, i.e. $E(b)=L_{\text{type}(b)}{\left(\text{length}(b)-b_{0}\right)}^{2}$, where $b_{0}$ is the resting length and L is the strength of that interaction (L varies according to bond type). The energy of an angle ϕ was similarly calculated using the ‘harmonic’ angle style, i.e. $E(\phi )=L_{type(\phi )}{\left(\phi -\phi_{0}\right)}^{2}$, where $\phi_{0}$ is the resting angle and k is again the interaction strength, and L again depends on the angle type of $\phi^{3}$ The resting lengths and angles for all energetic interactions were calculated using the resting geometry of our microtubule graph $G_{mt}$: a LAMMPS script was used to print the value of every angle interaction in the model, and these were collected and grouped based on value (all 153° angles, all 102° angles, etc). Each strength parameter was varied over the values in {3.0,9.0,18.0,30.0,39.0,48.0,57.0}, producing 7^5^ parameter combinations. Langevin dynamics were used, but with small temperature, to ensure stability and emphasize mechanical interactions. See table 1 and figure 3 for details on each strength parameter. See figure 4 for an illustration of varying resting positions and final energies as a result of varying these interaction parameters.

**Figure 2.**
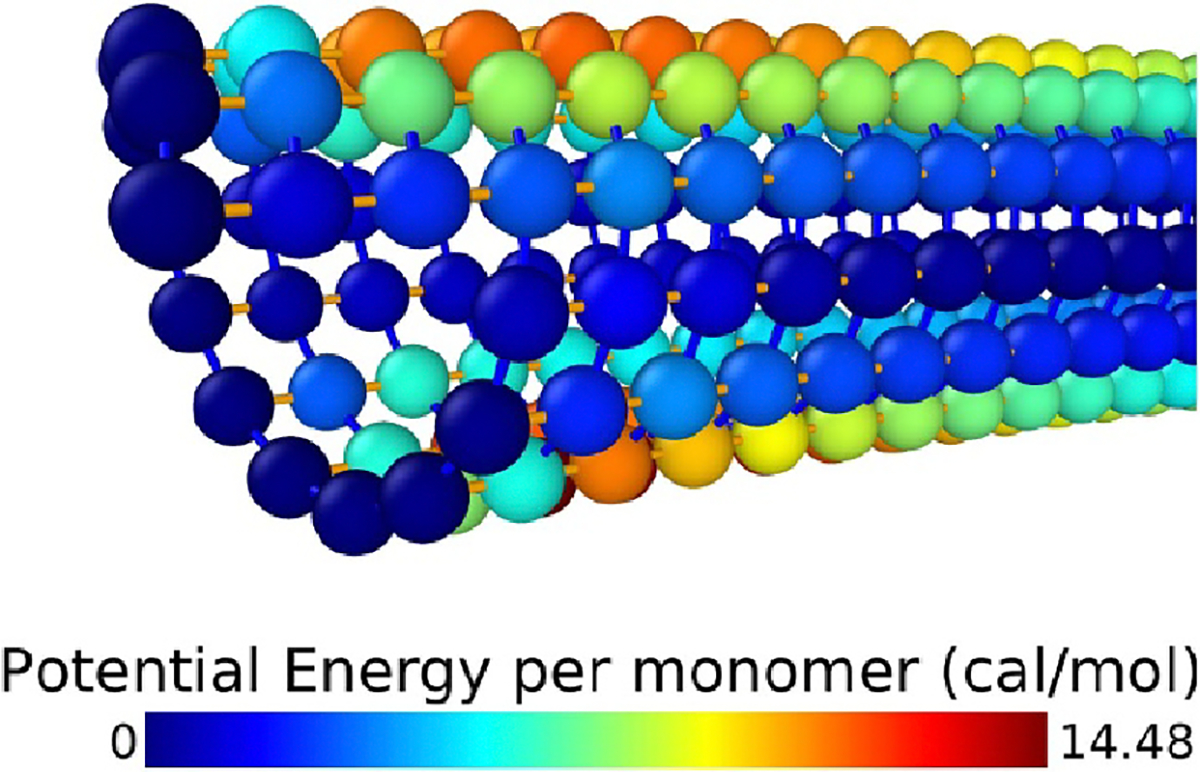
Microtubule model under bending load. Color of each particle indicates the sum of that particle’s share of all of the energetic interactions in which it participates. This view is on the claimed end; the other end, out of view, has a constant force applied. The flexural rigidity (EI) we measure from the stiffest MTs we simulate is within the (broad) range of values found by prior work for taxol-stabilized MTs (both simulated and measured; see Kikumoto et al (2006), Takasone et al (2002), VanBuren et al (2005)).

**Figure 3.**
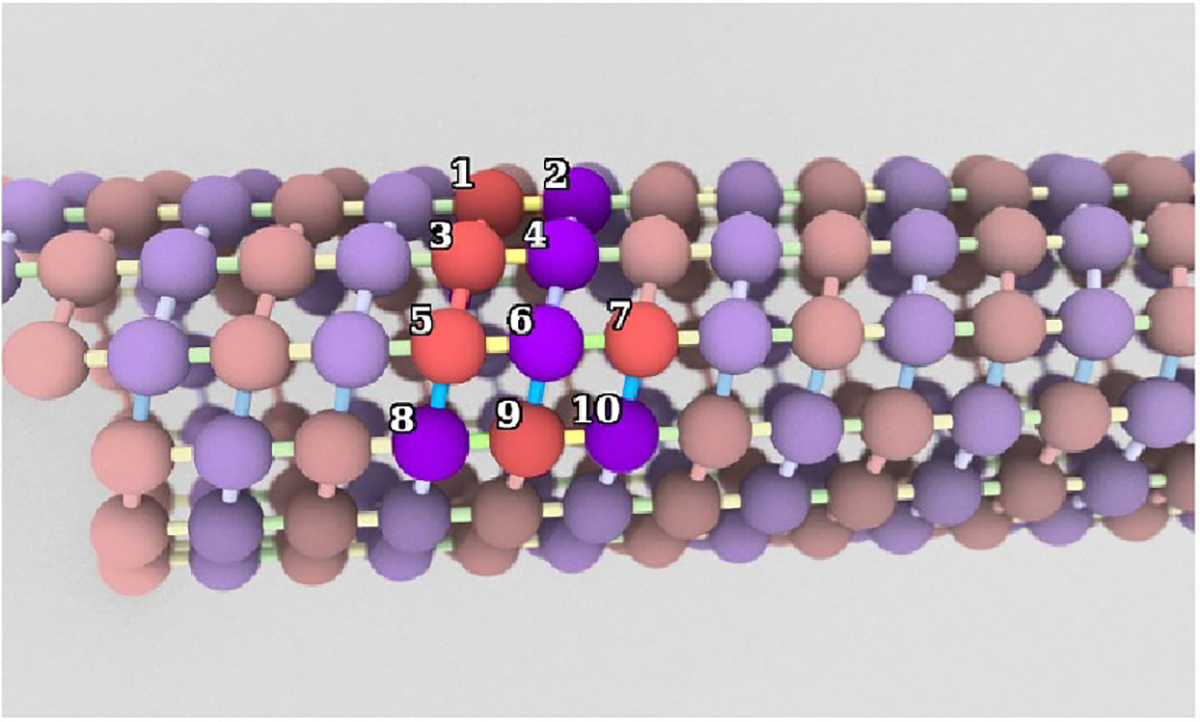
Microtubule model structure. Red spheres represent α-tubulin; purple spheres represent β-tubulin. Highlighted atoms at center are labeled to show example energetic interactions: each type of interaction indicated in table 1 (using the particle labels in this image) is applied everywhere in the model where that arrangement of particle and association types occurs in that position.

**Figure 4.**
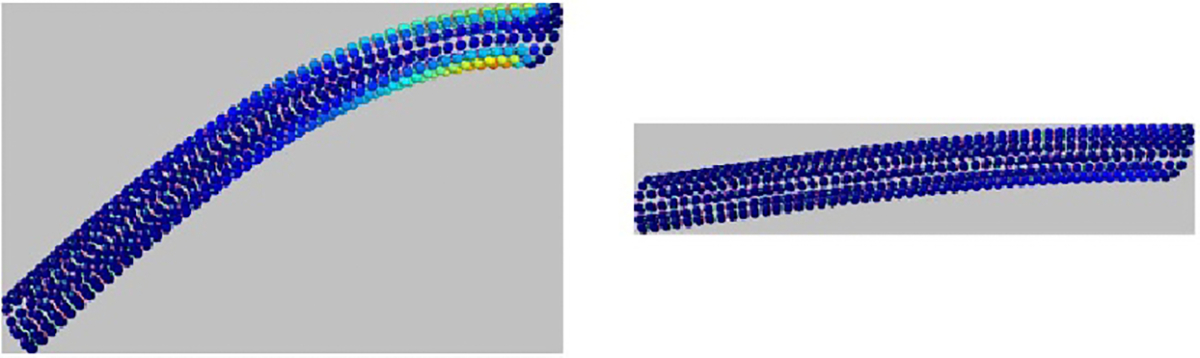
Changes in stiffness of microtubule model under constant load, as parameters controlling interaction strength are varied. We see qualitative differences in behavior as spring constants are adjusted between 0.1 and 1.9. The left and right images show the final timestep of simulations where all spring constants were set to the minimum and maximum strength, respectively. Particles (tubulin monomers) are colored according to their contribution to total potential energy of the configuration, identically to figure 2. All pictures show the microtubule at rest e.g. at the end of the simulation run using that parameter set.

GNU Parallel (Tange 2011) was used to run a simulation for each combination of interaction parameters, using the particle dynamics simulation engine LAMMPS. In each simulation, we clamp the first two rings of tubulin monomers (nodes 1–26) in place, and apply force (in the negative y direction) to the final two rings of monomers (nodes 599–624). This force starts at 0 and ramps up during the first 128000 timesteps (one step =0.018 ns) to its maximum value of 9×10^−14^ N. Once maximum force is reached, the simulation runs for 256000 additional timesteps, which in our experience was long enough for all particles to come to rest. See figure 2 for an illustration (visualized with Stukowski (2010)) of the potential energy per-particle at the final frame of a typical simulation run. Every K=32000 timesteps, we save the following for every particle: the position x, y, z; components of velocity $v_{x}$, $v_{y}$, $v_{z}$; components of force $F_{x}$, $F_{y}$, $F_{z}$; and the potential energy of the particle E. The dataset is then a concatenation of the 12 saved frames from every simulation run, comprising all combinations of input parameter values, where for each frame we have:

$x_{i}$, the input graph signal, a 624×10 matrix holding the position and velocity of each particle, as well as values of the four interaction coefficients; and

$y_{i}$, the output graph signal, a 624×1 matrix holding the potential energy calculated for each particle.

We note here that none of the inputs to the model encode information about any of the statistics of the system as a whole (for example, the total energy, the temperature or density of the surrounding solvent, etc). This was not necessary in our example simulations because these factors did not vary in our experiment. A more detailed data input would likely be necessary for our model to be implemented in a more complicated simulation scenario that tuned any of these system quantities between runs.

During training, after a training/validation split, we normalize the data by taking the mean and standard deviation of the $N_{\text{train}}\times 624\times 10$ input and $N_{\text{train}}\times 624\times 1$ output tensors along their first axis. Each data tensor is then reduced by the mean and divided by the standard deviation so that all 624×10 inputs to the network have zero mean and unit standard deviation. We normalize using the training data only.

### 3.2. Efficient calculation of GDD

**Figure 5.**
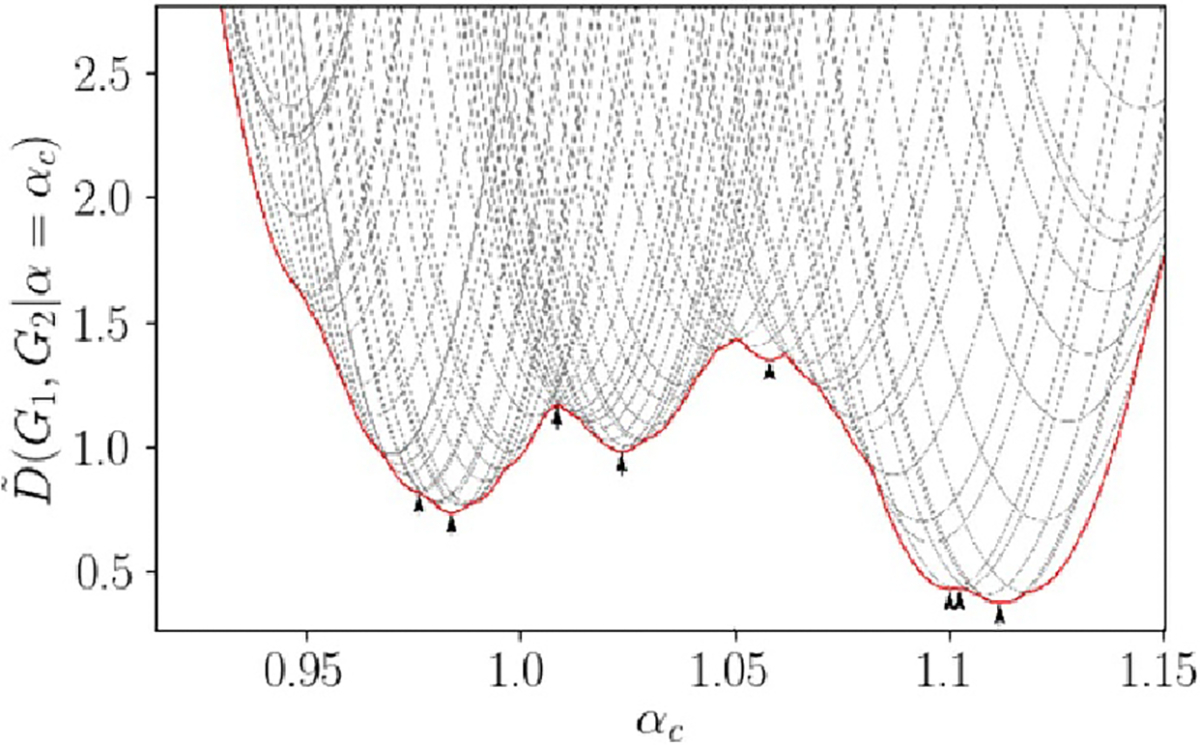
Plot of Linear Graph Diffusion Distance between two small random graphs, as α is varied. Each gray curve shows the objective function when P is fixed, as a function of α, and each curve represents a P matrix which is optimal at any value of α in the plotted range. The red curve shows the lower convex hull of all gray curves. Note that it is continuous but has discontinuous slope. Black arrows represent local optima. The discontinuous slope and high number of local optima illustrate why optimizing this function using univariate search over α is inefficient.

The joint optimization given in the definition of Linear GDD (equation (1)) is a nested optimization problem. If we set

$$\begin{array}{l} f(\alpha )=D\left(G_{1},G_{2}|\alpha \right)\\ \;=\underset{P|𝒞(P)}{inf}{\left\|\frac{1}{\alpha }PL\left(G_{1}\right)-\alpha L\left(G_{2}\right)P\right\|}_{F}, \end{array}$$

then each evaluation of f requires a full optimization of the matrix P subject to constraints 𝒞. When $L\left(G_{1}\right)$ and $L\left(G_{2}\right)$ are Graph Laplacians, f(α) is continuous, but with discontinuous derivative, and has many local minima (see figure 5). As a result, the naive approach of optimizing f(α) using a univariate optimization method like Golden section Search is inefficient. In this section we briefly describe a procedure for performing this joint optimization more efficiently. For a discussion of variants of the LGDD, as well as the theoretical justification of this algorithm, see Scott and Mjolsness (2019b).

First, we note that by making the constraints on P more restrictive, we upper-bound the original distance:

(3) $$\begin{array}{l} D\left(G_{1},G_{2}\right)=\underset{\begin{array}{c} P|𝒞(P)\\ \alpha >0 \end{array}}{inf}{\left\|\frac{1}{\alpha }PL\left(G_{1}\right)-\alpha L\left(G_{2}\right)P\right\|}_{F}\\ \;\leq \underset{\begin{array}{c} P|𝒮(P)\\ \alpha >0 \end{array}}{inf}{\left\|\frac{1}{\alpha }PL\left(G_{1}\right)-\alpha L\left(G_{2}\right)P\right\|}_{F}. \end{array}$$

In our case, 𝒞(P) represents orthogonality. As a restriction of our constraints we specify that P must be related to a *subpermutation* matrix (an orthogonal matrix having only 0 and 1 entries) $\tilde{P}$ as follows: $P=U_{2}\tilde{P}U_{1}^{T}$, where the $U_{i}$ are the fixed matrices which diagonalize $L\left(G_{i}\right):L\left(G_{i}\right)=U_{i}\Lambda_{i}U_{i}^{T}$. Then,

$$\begin{array}{l} D\left(G_{1},G_{2}\right)\leq \underset{\begin{array}{c} P|𝒮(P)\\ \alpha >0 \end{array}}{inf}{\left\|\frac{1}{\alpha }PL\left(G_{1}\right)-\alpha L\left(G_{2}\right)P\right\|}_{F}\\ \;=\underset{\begin{array}{c} \tilde{P}|subperm(\tilde{P})\\ \alpha >0 \end{array}}{inf}\|\frac{1}{\alpha }U_{2}\tilde{P}U_{1}^{T}U_{1}\Lambda_{1}U_{1}^{T}\\ \;-\alpha U_{2}\Lambda_{2}U_{2}^{T}U_{2}\tilde{P}U_{1}^{T}{\|}_{F}\\ \;=\underset{\begin{array}{c} \tilde{P}|subperm(\tilde{P})\\ \alpha >0 \end{array}}{inf}{\left\|\frac{1}{\alpha }U_{2}\tilde{P}\Lambda_{1}U_{1}^{T}-\alpha U_{2}\Lambda_{2}\tilde{P}U_{1}^{T}\right\|}_{F}\\ \;=\underset{\begin{array}{c} \tilde{P}|subperm(\tilde{P})\\ \alpha >0 \end{array}}{inf}{\left\|U_{2}\left(\frac{1}{\alpha }\tilde{P}\Lambda_{1}-\alpha \Lambda_{2}\tilde{P}\right)U_{1}^{T}\right\|}_{F}. \end{array}$$

Because the $U_{i}$ are rotation matrices (under which the Frobenius norm is invariant), this further simplifies to

$$D\left(G_{1},G_{2}\right)\leq \underset{\begin{array}{c} \tilde{P}|subperm(\tilde{P})\\ \alpha >0 \end{array}}{inf}{\left\|\frac{1}{\alpha }\tilde{P}\Lambda_{1}-\alpha \Lambda_{2}\tilde{P}\right\|}_{F}.$$

Furthermore, because the $\Lambda_{i}$ are diagonal, this optimization is equivalent to a rectangular linear assignment problem (RLAP) (Bijsterbosch and Volgenant 2010), between the diagonal entries $\lambda_{j}^{\left(1\right)}$ and $\lambda_{l}^{\left(2\right)}$ of $\Lambda_{1}$ and $\Lambda_{2}$, respectively, with the α-dependent cost of an assignment given by:

$$c_{\alpha }(\lambda_{j}^{\left(1\right)},\lambda_{l}^{\left(2\right)})={\left(\frac{1}{\alpha }\lambda_{j}^{\left(1\right)}-\alpha \lambda_{l}^{\left(2\right)}\right)}^{2}.$$

RLAPs are extensively studied. We use the general LAP solving package lapsolver (Heindl 2018) to comute $\tilde{P}$. In practice (and indeed in this paper) we set often set α=1, in which case the solution $\tilde{P}$ of the RLAP only acts as a preconditioner for the orthogonally-constrained optimization over P More generally, when alpha is allowed to vary (and therefore many RLAPs must be solved), a further speedup is attained by re-using partial RLAP solutions from previously-tested values of α to find the optimal assignment at $\alpha^{\prime }$. We detail how this may be done in out recent work (Scott and Mjolsness 2019b).

For the P matrices used in the experiments in this work, we set α=1 and used lapsolver to find an optimal assignment $\tilde{P}$. We then initialized an orthogonally-constrained optimization of 1 with $P=U_{2}\tilde{P}U_{1}^{T}$. This constrained optimization was performed using Pymanopt (Townsend et al 2016).

### 3.3. Graph coarsening

In this section we outline a procedure for determining the coarsened structure matrices to use in the hierarchy of GCN models comprising a GPCN. We use our microtubule graph as an example. In this case, we have two a-priori guidelines for producing the reduced-order graphs: (1) the reduced models should still be a tube and (2) it makes sense from a biological point of view to coarsen by combining the α-β pairs into single subunits. Given these restrictions, we can explore the space of coarsened graphs and find the coarse graph which is nearest to our original graph (under the GDD).

**Figure 6.**
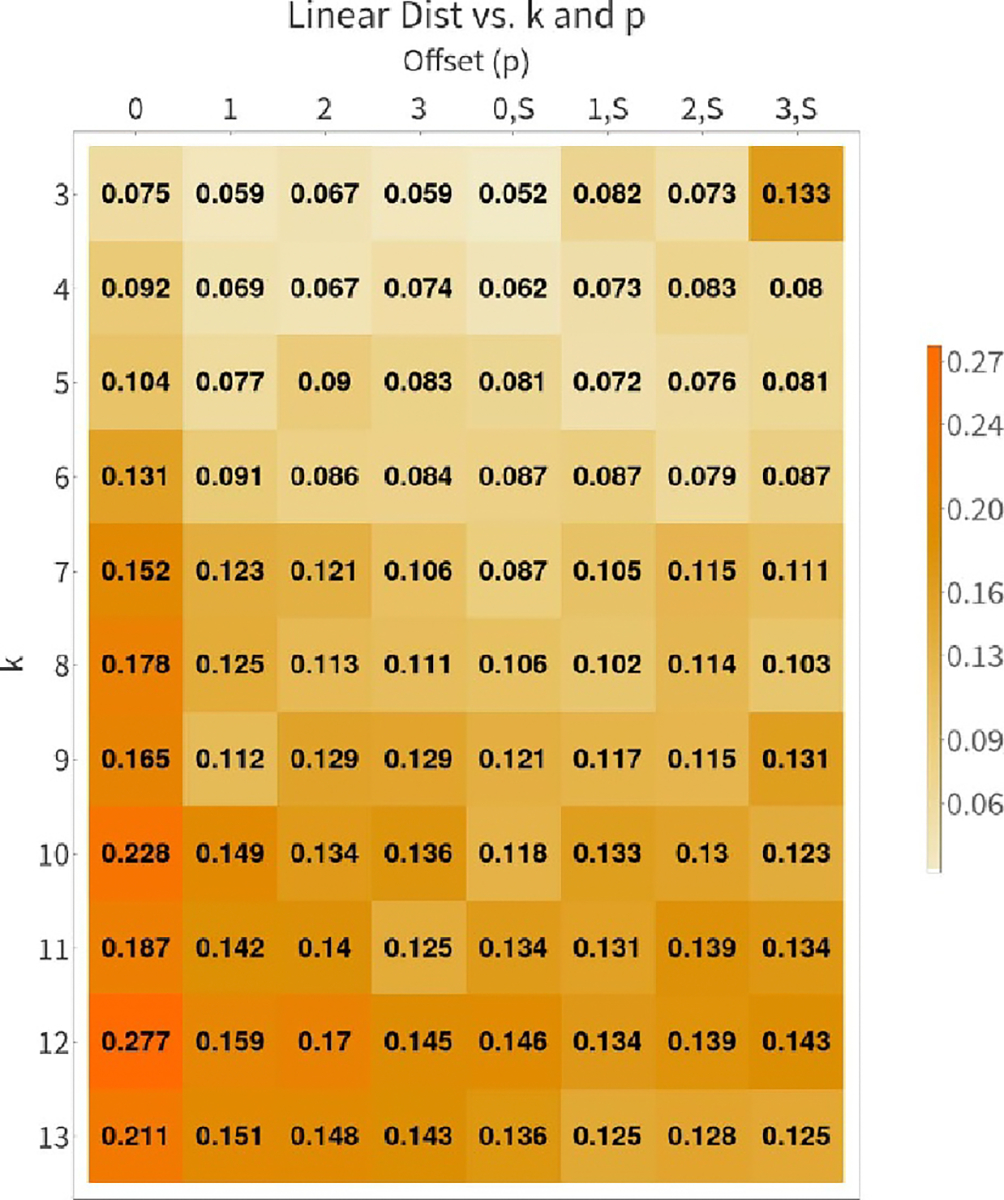
Directed Graph Diffusion Distance (GDD) between offset tube graphs and $G_{mt}$. Table cells colored by value. We see from this comparison that the two graphs which are closest to $G_{mt}$ are $G_{\text{Tube}(24,3,0)}$ and $G_{\text{Tube}(24,3,0)}$ with an edge weight of 2 for connections along the seam, motivating our choice of $G_{\text{Tube}(24,3,0)}$ (unweighted) as the coarsest graph in our hierarchy.

**Figure 7.**
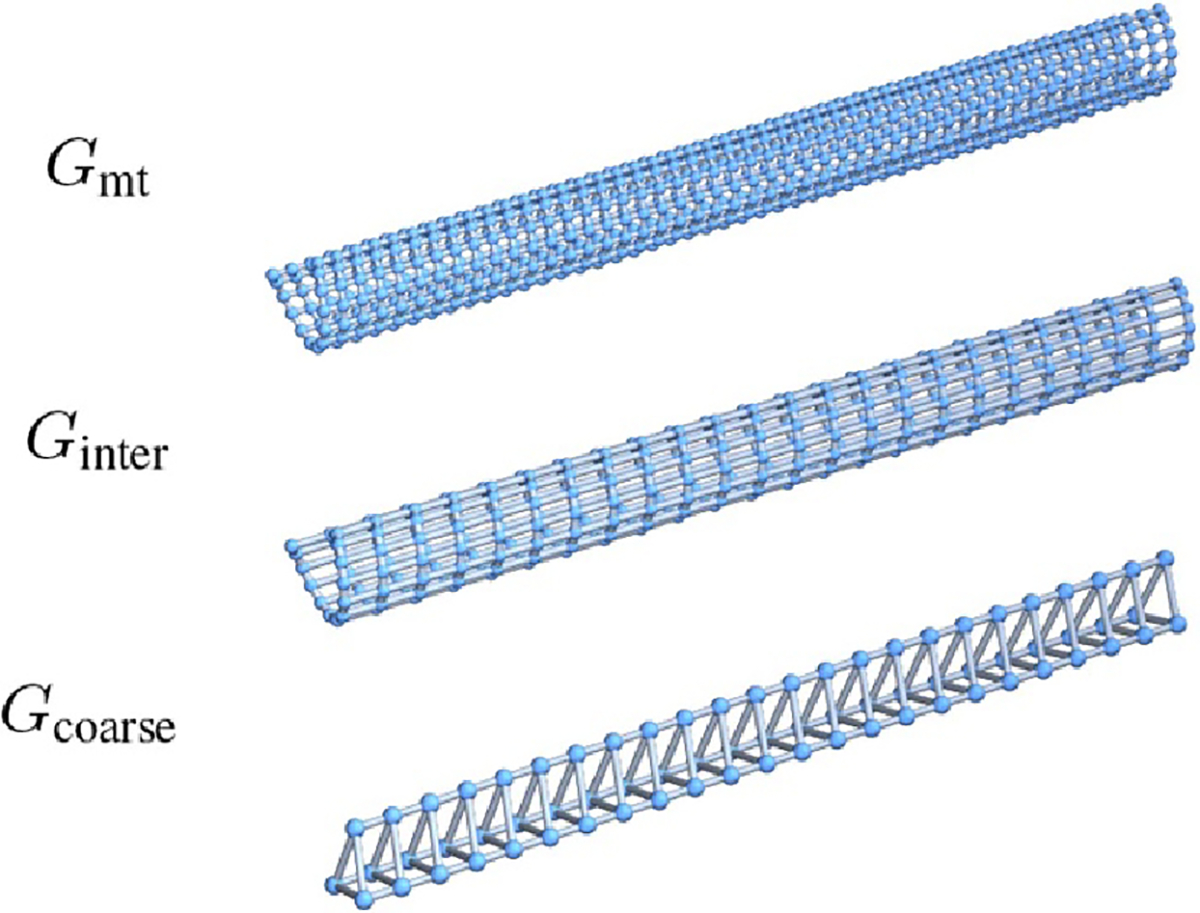
Three graphs used to create structure matrices for our GPCN model. Top: microtubule graph. Center: Offset tube with 13 subunits per turn, length 24, and offset 1. Bottom: Tube with 3 subunits per turn, no offset, and length 24.

Our microtubule model is a tube of length 48 units, 13 units per complete ‘turn’, and with the seam offset by three units. We generalize this notion as follows: Let p be the offset, and k be the number of monomers in one turn of the tube, and n the number of turns of a tube graph $G_{\text{Tube}(n,k,p)}$. The graph used in our simulation is thus $G_{mt}=G_{\text{Tube}(48,13,3)}$. We pick the medium scale model $G_{\text{inter}}$ to be $G_{\text{Tube}(24,13,1)}$, as this is the result of combining each α-β pair of tubulin monomer units in the fine scale, into one tubulin dimer unit in the medium scale. We pick the coarsest graph $G_{\text{coarse}}$ by searching over possible offset tube graphs. Namely, we vary k∈{3,4,…12} and p∈{0,1,2,3}, and compute the optimal $P^{*}$ and its associated distance $D(G_{\text{Tube}(24,k,p)},G_{mt}|P=P^{*})$. Figure 6 shows the distance between $G_{mt}$ and various other tube graphs as parameters p and k are varied. The nearest $G_{\text{Tube}(24,k,p)}$ to $G_{mt}$ is that with p=0 and k=3. Note that figure 6 has two columns for each value of k: these represent the coarse edges along the seam having weight (relative to the other edges) 1 (marked with an S) or having weight 2 (no S). This is motivated by the fact that our initial condensing of each dimer pair condensed pairs of seam edges into single edges. Figure 7 illustrates the resulting fine-scale, medium-scale, and coarse-scale graph structures.

## 4. Machine learning experiments

### 4.1. Experimental procedure

This section contains several experiments comparing our model, and its variants, to other types of GCNs. All models were trained using ADAM with default hyperparameters, in TensorFlow (Abadi et al 2016). Random seeds for Python, TensorFlow, Numpy, and Scipy were all initialized to the same value for each training run, to ensure that the train/validation split is the same across all experiments, and the batches of drawn data are the same. See supplementary material for version numbers of all software packages used. Training batch size was set to 8, all GCN layers have ReLU activation, and all dense layers have sigmoidal activation with the exception of the output layer of each network (which is linear). All modes were trained for 1000 epochs of 20 batches each. The time per batch of each model is listed in table 4.

Since hardware implementations may differ, we estimate the computational cost in Floating Point OPerations (FLOPs) of each operation in our models. The cost of a graph convolutional layer with n×n structure matrix Z,n×F input data X, and F×C filter matrix W is estimated as: nF(|Z|+C), where |Z| is the number of nonzero entries of Z. This is calculated as the sum of the costs of the two matrix multiplications X⋅W and Z⋅XW, with the latter assumed to be implemented as sparse matrix multiplication and therefore requiring O(|Z|nF) operations. For implementation reasons, our GCN layers (across all models) do not use sparse multiplication; if support for arbitrary-dimensional sparse tensor outer products is included in TensorFlow in the future, we would expect the wall-clock times in table 4 to decrease. The cost of a dense layer (with n×F input data X, and F×C filter matrix W) applied to every node separately is estimated as: O(nFC). The cost of taking the dot product between a n×k matrix and a k×m matrix (for example, the restriction/prolongation by P) is estimated as O(nmk).

For GPCN models, P matrices were calculated using Pymanopt (Townsend et al 2016) to optimize equation (1) subject to orthogonality constraints. The same P were used to initialize the (variable) P matrices of A-GPCN models.

### 4.2. Evaluation of GPCN variants

Our proposed model uses a hierarchy of GCNs to predict energy of a molecule at several spatial scales. The computational cost of a graph convolutional layer is approximately quadratic in the number of nodes in the underlying graph. We would therefore expect to see efficiency gains when some number of graph convolution layers are operating on a reduced graph. In this subsection we present numerical experiments showing that this is indeed the case: the accuracy gained (per unit of computational expenditure) is higher for deeper hierarchies. Additionally, the adaptive model(s) universally outperform their non-adaptive counterparts.

We compare the following versions of our model:

- a two-level GPCN with static P-matrices;
- a three-level GPCN with static P-matrices;
- both of the above, but with P matrices allowed to vary during training (adjusted with the same backpropagation signals which are used to modify the convolution weights).

**Figure 8.**
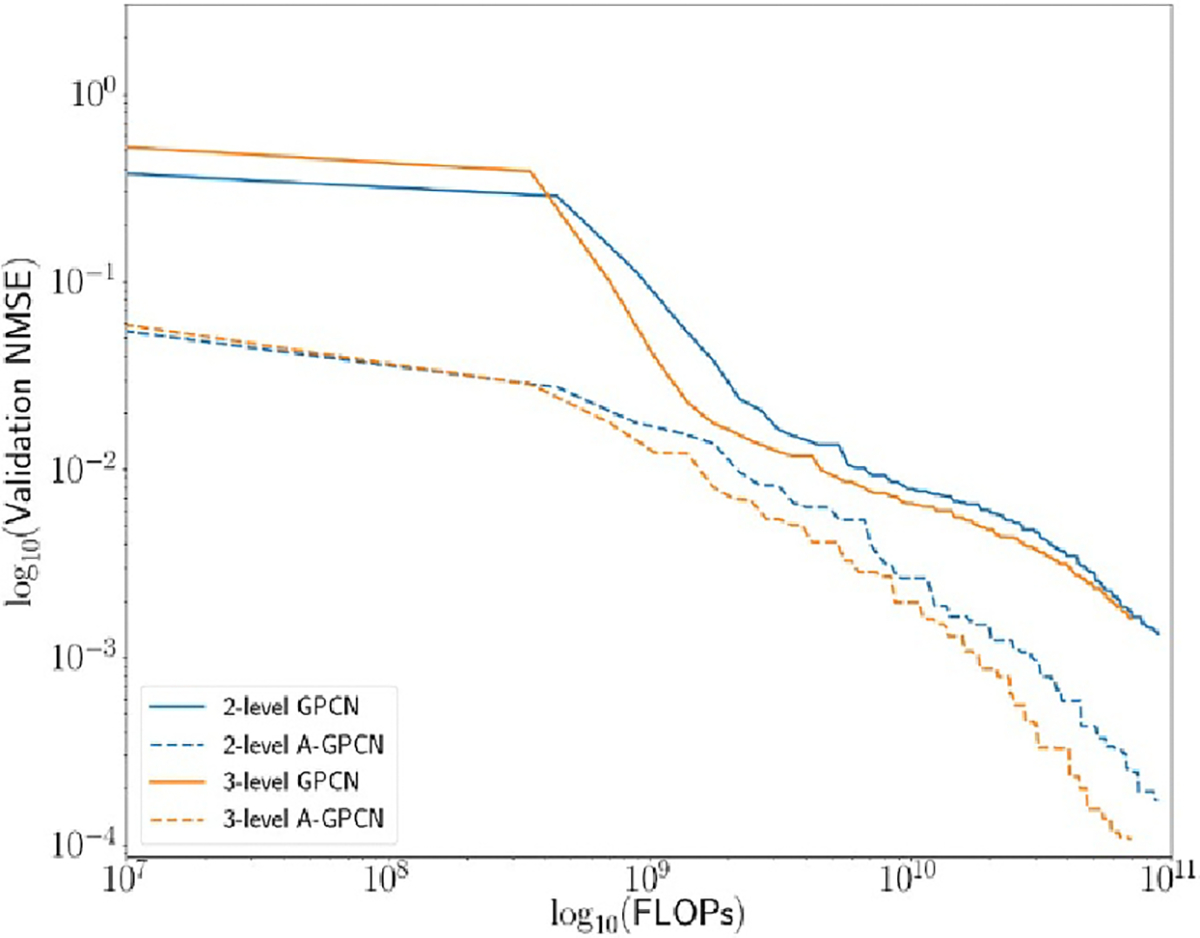
Comparison of mean squared error (MSE) on held-out validation data (normalized by averaging over the validation set) as a function of FLOPs expended, for variants of our model. We see that the adaptive and non-adaptive models occupy separate regimes (the adaptive models are superior), and within each the depth-3 model outperforms the depth-2 one.

Figure 8 and table 3 summarize these results.

### 4.3. Evaluation of training schedules

In contrast to the prior section, where we use the same training strategy and evaluate the efficiency of different variants of our model, in this section we fix the model architecture and evaluate the effect of different training schedules. Specifically, we compare the computational cost of training the entire GPCN at once, versus training the different ‘resolutions’ (meaning the different GCNs in the hierarchy) of the network according to a more complicated training schedule. This approach is motivated by recent work in coarse-to-fine training of both flat and CNNs (Scott and Mjolsness 2019a, Zhao et al 2019, Haber et al 2018, Dou and Wu 2015, Ke et al 2017), as well as the extensive literature on algebraic multigrid (AMG) methods (Vanek et al 1996).

AMG solvers for differential equations on a mesh (which arises as the discretization of some volume to be simulated) proceed by performing numerical ‘smoothing steps’ at multiple resolutions of discretization. The intuition behind this approach is that modes of error should be smooth at a spatial scale which is equivalent to their wavelength, i.e. the solver should not spend many cycles resolving long-wavelength errors at the finest scale, since they can be resolved more efficiently at the coarse scale. Given a solver and a hierarchy of discretizations, the AMG literature defines several types of training procedures or ‘cycle’ types (F-cycle, V-cycle, W-cycle). These cycles can be understood as being specified by a recursion parameter γ, which controls how many times the smoothing or training algorithm visits all of the coarser levels of the hierarchy in between smoothing steps at a given scale. For example, when γ=1 the algorithm proceeds from fine to coarse and back again, performing one smoothing step at each resolution—a ‘V’ cycle.

We investigate the efficiency of training 3-level GPCN and A-GPCN (as described in section 4.2), using multigrid-like training schedules with γ∈{0,1,2,3}, as well as ‘coarse-to-fine’ training: training the coarse model to convergence, then training the coarse and intermediate models together (until convergence), then finally training all three models at once. Error was calculated at the fine-scale. For coarse-to-fine training convergence was defined to have occurred once 10 epochs had passed without improvement of the validation error.

**Figure 9.**
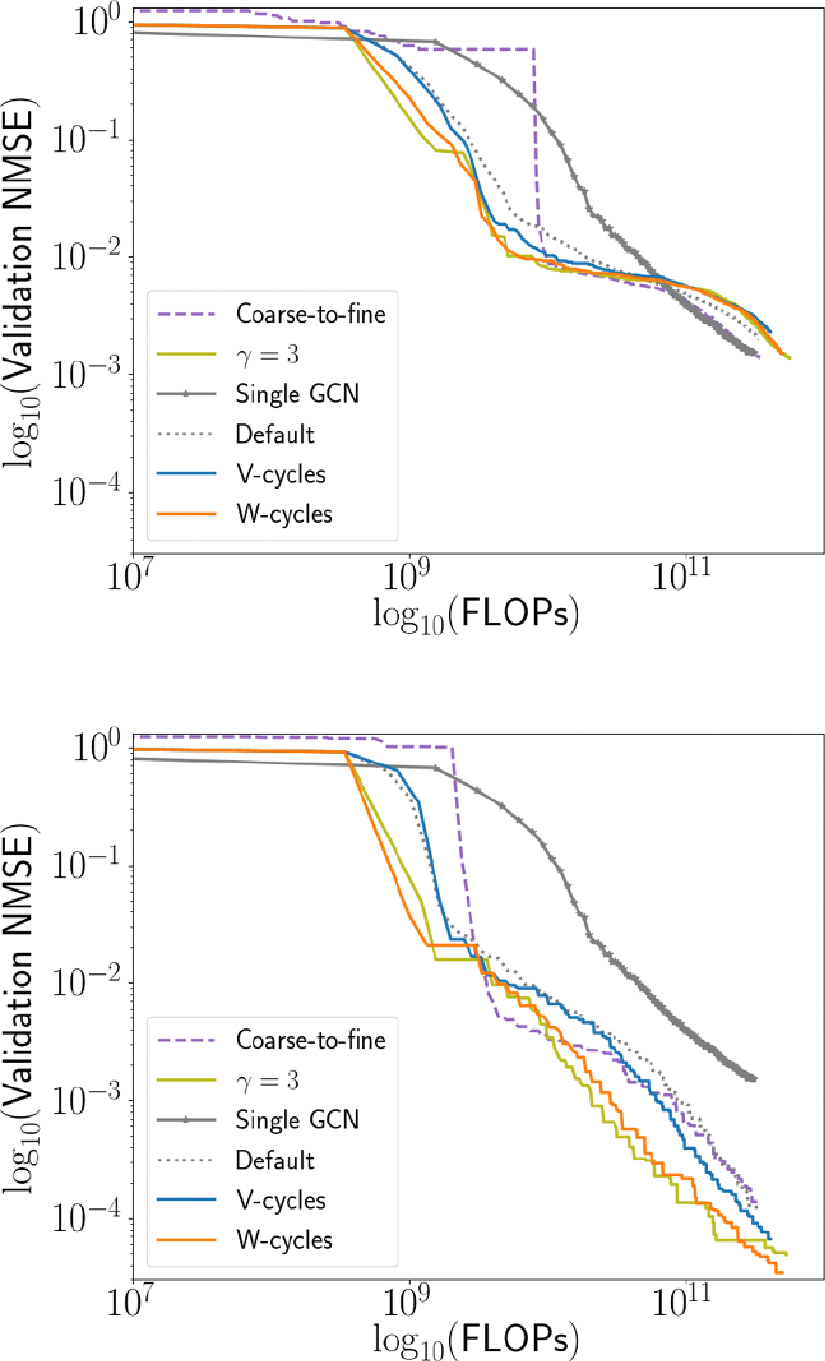
Effect of varying training schedule for training a GPCN model. Notably, The various multigrid training cycles result in models which are more accurate, and do so more efficiently. Top: FLOPs vs. NMSE for training GPCNs with multigrid training schedules. Bottom: same, but with A-GPCNs.

Our experiments (see figure 9) show that these training schedules do result in a slight increase in efficiency of the GPCN model, especially during the early phase of training. The increase is especially pronounced for the schedules with γ=2 and γ=3. Furthermore, these multigrid training schedules produce models which are more accurate than the GPCN and A-GPCN models trained in the default manner. As a final note, previous work (Scott and Mjolsness 2019a) has shown that these types of multiscale neural network architectures, with this type of multigrid training schedule may also be more efficient in a ‘statistical’ sense—that is, require much less data to find an equivalent or better local minimum of error. A third type of efficiency results from the fact that once trained, querying the machine learning model is faster than running an entire simulation. This means that the cost of generating the initial dataset and training the model is amortized over the time gained by using the machine learning model as an approximator. We would expect our model to also perform well under both of these latter measures of efficiency—one run of our fine-scale simulation took approximately 20 min, whereas querying the trained GPCN takes tenths of milliseconds. However, quantifying this possibility further is beyond the scope of this paper.

### 4.4. Comparison with DiffPool

Graph coarsening procedures are in general not differentiable. DiffPool (Ying et al 2018) aims to address this by constructing an auxiliary GCN, whose output is a pooling matrix. Formally: Suppose that layer l of a GCN we have a $n_{l}\times n_{l}$ structure matrix $Z^{\left(l\right)}$ and a n×F data matrix $X^{\left(l\right)}$. In addition to GCN layers as described in section 2, Ying et al define a pooling operation at layer l as:

$$S^{\left(l\right)}=\sigma \left({GCN}_{\text{pool}\;}\left(Z^{\left(l\right)},X^{\left(l\right)},{\left\{\theta_{1}^{\left(i\right)}\right\}}_{l=1}^{m}\right)\right)$$

where ${GCN}_{\text{pool}}$ is an auxillary GCN with its own set of parameters ${\left\{\theta_{1}^{\left(i\right)}\right\}}_{l=1}^{m}$, and σ is the softmax function. The output of ${GCN}_{\text{pool}}$ is a $n\times n_{\text{coarse}}$ matrix, each row of which is softmaxed to produce an affinity matrix S whose rows each sum to 1, representing each fine-scale node being connected to one unit’s worth of coarse-scale nodes. The coarsened structural and data matrices for the next layer are then calculated as:

(5) $$X^{\left(l+1\right)}=S^{{\left(l\right)}^{T}}X^{\left(l\right)}\;Z^{\left(l+1\right)}=S^{{\left(l\right)}^{T}}Z^{\left(l\right)}S^{\left(l\right)}$$

Clearly, the additional GCN layers required to produce $S^{\left(l\right)}$ incur additional computational cost. We compare our 3-level GPCN (adaptive and not) models from the experiment in section 4.5 to a model which has the same structure, but in which each P matrix is replaced by the appropriately-sized output of a DiffPOOL module, and furthermore the coarsened structure matrices are produced as in equation (5).

We see that our GPCN model achieves comparable validation loss with less computational work, and our A-GPCN model additionally achieves lower absolute validation loss.

### 4.5. Comparison to other GCN ensemble models

In this experiment we demonstrate the efficiency advantages of our model by comparing our approach to other ensemble GCNs. Within each ensemble, ours and others, each GCN model consists of several graph convolution layers, followed by several dense layers which are applied to each node separately (node-wise dense layers can be alternatively understood as a GCN layer with Z=I, although we implement it differently for efficiency reasons). The input to the dense layers is the node-wise concatenation of the output of each GCN layer. Each ensemble is the sum output of several such GCNs. We compare our models to 1, 2, and 3-member GCN ensembles with the same number of filters (but all using the original fine-scale structure matrix).

We also compare our model to the work of Abu-El-Haija et al (2018), who introduce the N-GCN model: an ensemble GCN in which each ensemble member uses a different power $Z^{r}$ of the structure matrix (to aggregate information from neighborhoods of radius r). We include a N-GCN with radii (1,2,4) and a N-GCN with radii (1,2,4,8,16).

**Figure 10.**
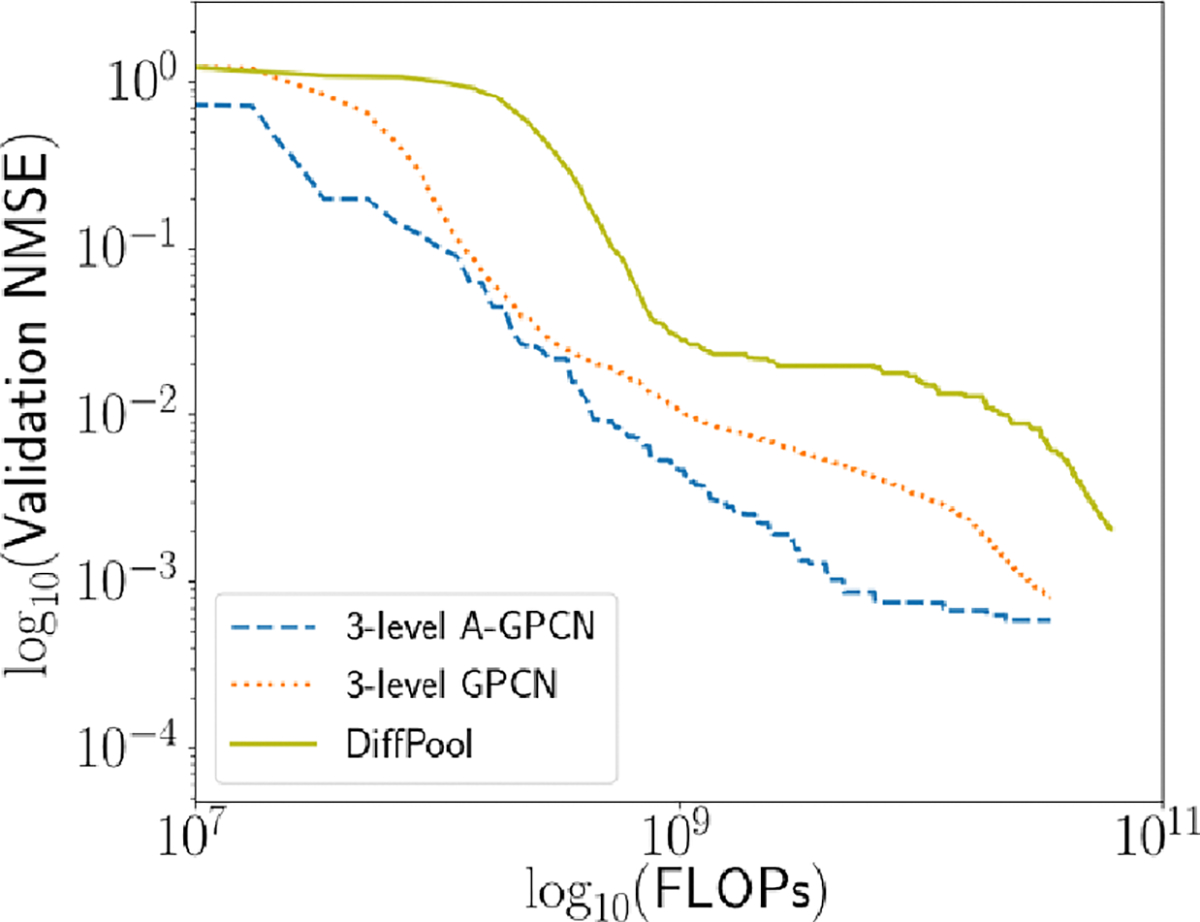
Comparison of 3-level GPCN and A-GPCN models to a 3-level GPCN which uses DiffPOOL modules to coarsen the input graph and data. Our models improve over DiffPOOL in terms of both efficiency and final error.

**Figure 11.**
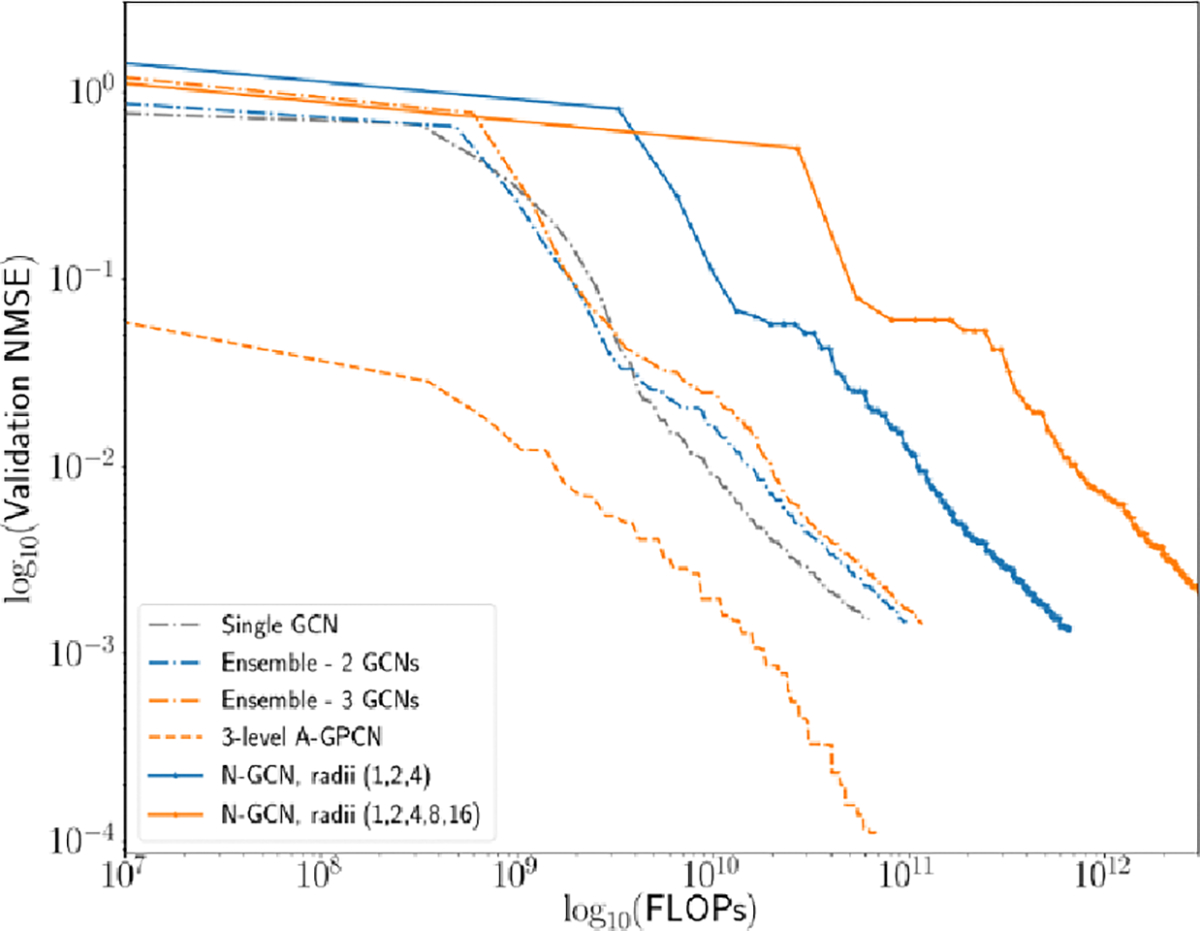
Comparison of Normalized MSE on held-out validation data as a function of FLOPs expended for a variety of ensemble Graph Convolutional Network Models. Plotted error is is the minimum validation error of the model over training thus far. We see that especially in early stages of training, our model formulation learns faster (e.g. requires fewer FLOPs) than an ensemble of 2, 3 or 5 GCNs with the same number of filters.

We summarize the structure of each of our models in table 2. In figure 11 we show a comparison between each of these models, for one particular random seed (42). Error on the validation set is tracked as a function of computational cost expended to train the model (under our cost assumption given above). We see that all four GPCN models outperform the other types of ensemble model during early training, in the sense that they reach lower levels of error for the same amount of computational work performed. Additionally, the adaptive GPCN models outperform all other models in terms of absolute error: after the same number of training epochs (using the same random seed) they reach an order of magnitude lower error. Table 3 shows summary statistics for several runs of this experiment with varying random seeds; we see that the A-GPCN models consistently outperform all other models considered. Note that figures 11,10, and 9 plot the normalize mean squared error. This unitless value compares the output signal to the target after both are normalized by the procedure described in section 3.1.

### 4.6. Machine learning summary

The machine learning model presented in section 2.4 is validated through numerical experiments on an evaluation dataset. First, variations of our architecture are compared in section 4.2, demonstrating that deeper versions of this architecture perform significantly better, and that re-training the P matrices leads to further accuracy gains. In section 4.3, we fix the model architecture to be the best-performing of those considered in section 4.2, and examine the effect of varying training schedules, including multigrid-like and coarse-to-fine training. These experiments demonstrate that our model achieves comparable error in less computation when trained in a multigrid fashion. Finally in sections 4.4 and 4.5, we validate our model by training other types of GCN models on the same learning task. We show significant accuracy gains over previous GCN ensemble models such as Abu-El-Haija et al (2018) and also outperform DiffPool (Ying et al 2018), which learns pooling maps during the training process. All results comparing our model to other GCN models are summarized in tables 3 and 4. Together these experiments demonstrate the superior accuracy and efficiency of our machine learning architecture.

## 5. Future work

### 5.1. Differentiable models of MD

This work demonstrates the use of feed-forward neural networks to approximate the energetic potentials of a mechanochemical model of an organic molecule. Per-timestep, GCN models may not be as fast as highly-parallelized, optimized MD codes. However, neural networks are highly flexible function approximators: the GCN training approach outlined in this paper could also be used to train a GCN which predicts the energy levels per particle at the end of a simulation (once equilibrium is reached), given the boundary conditions and initial conditions of each particle. In the case of our MT experiments, approximately 3×10^5^ steps were required to reach equilibrium. The computational work to generate a suitably large and diverse training set would then be amortized by the GCN’s ability to generalize to initial conditions, boundary conditions, and hyperparameters outside of this data set. Furthermore, this GCN reduced model would be fully differentiable, making it possible to perform gradient descent with respect to any of these inputs. In particular, we derive here the gradient of the input to a GCN model with respect to its inputs.

#### 5.1.1. Derivation of energy gradient w.r.t position

As described above, the output of our GCN (or GPCN) model is a n×1 matrix (or vector) Y, representing the energy of each simulated particle.. The total energy of the molecule at position X is given by a sum over monomers, $E=\sum_{i=1}^{n}[Y{]}_{i}$. Note that any GCN’s initial layer update is given by the update rule:

$$X‘=g_{1}\left(ZXW_{1}+b_{1}\right).$$

During backpropagation, as an intermediate step of computing the partial derivatives of energy with respect to $W_{1}$ and $b_{1}$, we must compute the partial $\frac{\partial E}{\partial A_{1}}$ of energy with respect to the input to the activation function $g_{1}$:

$$\begin{array}{l} A_{1}=ZXW_{1}+b_{1}\\ X‘=g_{1}\left(A_{1}\right). \end{array}$$

We therefore assume we have this derivative. By the Chain Rule for matrix derivatives:

$${\left[\frac{\partial E}{\partial X}\right]}_{ij}=\frac{\partial E}{\partial [X{]}_{ij}}=\sum_{k,p}\frac{\partial E}{\partial {\left[A_{1}\right]}_{kp}}\frac{\partial {\left[A_{1}\right]}_{kp}}{\partial \left[x_{ij}\right]}.$$

Since

$${\left[A_{1}\right]}_{kp}=\left(\sum_{c,d}{\left[Z\right]}_{kc}{\left[X\right]}_{cd}{\left[W_{1}\right]}_{dp}\right)+{\left[b_{1}\right]}_{kp}$$

and therefore

(6) $$\begin{array}{l} \frac{\partial {\left[A_{1}\right]}_{kp}}{\partial {\left[X\right]}_{ij}}={\left[Z\right]}_{ki}{\left[W_{1}\right]}_{jp},\\ \;\frac{\partial E}{\partial {\left[X\right]}_{ij}}=\sum_{k,p}\frac{\partial E}{\partial {\left[A_{1}\right]}_{kp}}{\left[Z\right]}_{ki}{\left[W_{1}\right]}_{jp}\\ \;\frac{\partial E}{\partial X}=Z^{T}\frac{\partial E}{\partial A_{1}}W_{1}^{T}. \end{array}$$

Furthermore, since our GPCN model is a sum of the output of several GCNs, we can also derive a backpropagation equation for the gradient of the fine-scale input, X, with respect to the energy prediction of the entire ensemble. Let $E^{\left(i\right)}$ represent the total^4^ fine-scale energy prediction of the *i*th member of the ensemble, so that $E=\sum_{i=1}^{k}E^{\left(i\right)}$. Then, let

$$\frac{\partial E^{\left(i\right)}}{\partial X^{\left(i\right)}}=Z^{{\left(i\right)}^{T}}\frac{\partial E^{\left(i\right)}}{\partial A_{1}^{\left(i\right)}}W_{1}^{{\left(i\right)}^{T}}$$

be the application of equation (6) to each GCN in the ensemble. Since the input to the ith member of the ensemble is given by $X^{\left(i\right)}=P_{1,i}^{T}X$, we can calculate the gradient of $E^{\left(i\right)}$ with respect to X, again using the Chain Rule:

$$\frac{\partial E^{\left(i\right)}}{\partial [X{]}_{mn}}=\sum_{s=1}^{N_{s}}\sum_{t=1}^{N_{t}}\frac{\partial E^{\left(i\right)}}{\partial {\left[X^{\left(i\right)}\right]}_{st}}\frac{\partial {\left[X^{\left(i\right)}\right]}_{st}}{\partial [X{]}_{mn}}\;\;=\sum_{s=1}^{N_{s}}\sum_{t=1}^{N_{t}}\frac{\partial E^{\left(i\right)}}{\partial {\left[X^{\left(i\right)}\right]}_{st}}\frac{\partial {\left[P_{1,i}^{T}X\right]}_{st}}{\partial [X{]}_{mn}}\;\;=\sum_{s=1}^{N_{s}}\sum_{t=1}^{N_{t}}\frac{\partial E^{\left(i\right)}}{\partial {\left[X^{\left(i\right)}\right]}_{st}}\delta_{tm}{\left[P_{1,i}\right]}_{ns}\;\;=\sum_{s=1}^{N_{s}}\frac{\partial E^{\left(i\right)}}{\partial {\left[X^{\left(i\right)}\right]}_{sm}}{\left[P_{1,i}\right]}_{ns}$$

Therefore,

$$\frac{\partial E^{\left(i\right)}}{\partial [X{]}_{mn}}=P_{1,i}\frac{\partial E^{\left(i\right)}}{\partial X^{\left(i\right)}}$$

and so

$$\frac{\partial E}{\partial X}=\sum_{i=1}^{k}\frac{\partial E^{\left(i\right)}}{\partial X}=\sum_{i=1}^{k}P_{1,i}\frac{\partial E^{\left(i\right)}}{\partial X^{\left(i\right)}}$$

This backpropagation rule may then be used to adjust X, and thereby find low-energy configurations of the molecular graph. Additionally, analogous to the GCN training procedure outlined in section 4.3, this optimization over molecule positions could start at the coarse scale and be gradually refined.

### 5.2. Tensor factorization

Recent work has re-examined GCNs in the context of the extensive literature on tensor decompositions. LanczosNet (Liao et al 2019), uses QR decomposition of the structure matrix to aggregate information from large neighborhoods of the graph. The ‘Tensor Graph Convolutional Network’ of Zhang et al 2018, is a different decomposition method, based on graph factorization; a product of GCNs operating on each factor graph can be as accurate as a single GCN acting on the product graph. Since recent work (Scott and Mjolsness 2019aa) has shown that the GDD of a graph product is bounded by the distances between the factor graphs, it seems reasonable to combine both ideas into a model which uses a separate GPCN for each factor. One major benefit of this approach would be that a transfer-learning style approach can be used. For example, we could train a product of two GCN models on a short section of microtubule; and then re-use the weights in a model that predicts energetic potentials for a longer microtubule. This would allow us to extend our approach to MT models whose lengths are biologically relevant, e.g. 10^3^ tubulin monomers.

### 5.3. Graph limits

Given that *in vivo* MTs are longer than the one simulated in this paper by a factor of as much as 200x, future work will focus on scaling these methods to the limit of very large graphs. In particular, this means repeating the experiments of Section 4, but with longer tube graphs. We hypothesise that tube graphs which are closer to the microtubule graph (under the LGDD) as their length n→∞ will be more efficient reduced-order models for a GPCN hierarchy. This idea is similar to the ‘graphons’ (which are the limits of sequences of graphs which are Cauchy under the Cut-Distance of graphs) introduced by Lovász 2012). To show that it is reasonable to define a ‘graph limit’ of microtubule graphs in this way, we plot the distance between successively longer microtubule graphs. Using the same notation as in section 3.3, we define three families of graphs:

- $G_{\text{Grid}}(n,13)$: Grids of dimensions n×13, and;
- $G_{\text{Tube}(n,13,1)}$: Microtubule graphs with 13 protofilaments, of length n, with offset 1, and;
- $G_{\text{Tube}(2n,13,3)}$: Microtubule graphs with 13 protofilaments, of length 2n, with offset 3.

**Figure 12.**
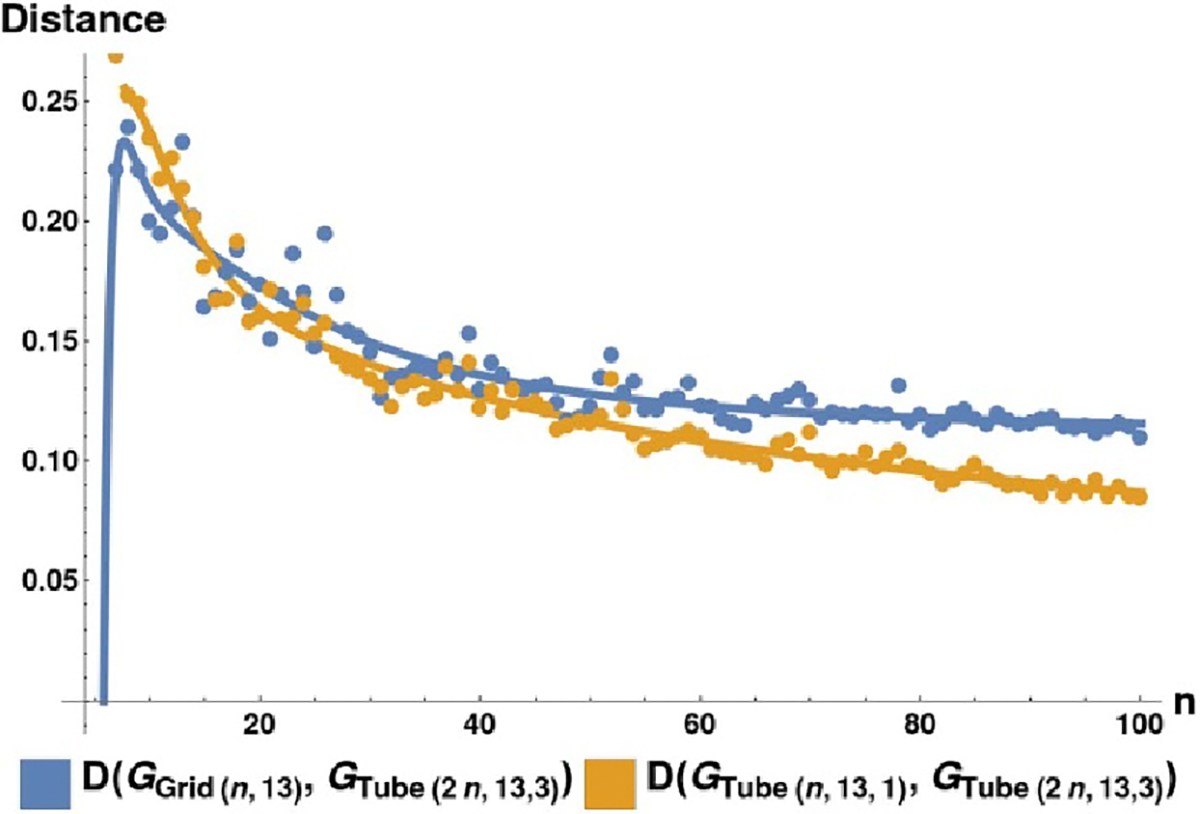
Limiting behavior of two classes of distances between graphs, as a function of graph size. We plot $D\left(G_{\text{Tube}(n,13,1)},G_{\text{Tube}(2n,13,3)}\right)$ and $D\left(G_{\text{Grid}(n,13)},G_{\text{Tube}(2n,13,3)}\right)$ as a function of n, along with seventh-degree polynomial fit curves of each. The smaller tube graphs are closer than the grid graphs to the larger tube, even in the large-graph limit.

In this preliminary example, as n is increased, we see a clear distinction in the distances $D\left(G_{\text{Tube}(n,13,1)},G_{\text{Tube}(2n,13,3)}\right)$ and $D\left(G_{\text{Grid}(n,13)},G_{\text{Tube}(2n,13,3)}\right)$, with the former clearly limiting to a larger value as n→∞ (see Figure 12).

## 6. Conclusion

We introduce a new type of graph ensemble model which explicitly learns to approximate behavior at multiple levels of coarsening. Our model outperforms several other types of GCN, including both other ensemble models and a model which coarsens the original graph using DiffPool. We also explore the effect of various training schedules, discovering that A-GPCNs can be effectively trained using a coarse-to-fine training schedule. We present the first use of GCNs to approximate energetic potentials in a model of a microtubule.

## Supplementary Material

Scott_Mjolsness_Code_Repo_REV

## Figures and Tables

**Table 1.** Description of energetic interactions in microtubule simulation, according to the labels in figure 3.

Association interactions			

Description	Examples	Resting Length	Strength Param.
Lateral association inside lattice	1, 3), (2, 4)	5.15639 nm	LatAssoc
Lateral association across seam	5, 8), (6, 9)	5.15639 nm	LatAssoc
Longitudinal association	1, 2), (3, 4)	5.0 nm	LongAssoc
Angle interactions			
Description	Examples	Resting Angle	Strength Param.
Pitch angle inside lattice	1, 3, 5), (2, 4, 6)	153.023°	LatAngle
Longitudinal angle	5, 6, 7), (8, 9, 10)	180°	LongAngle
Lattice cell acute angle	3, 4, 6), (3, 5, 6), (5, 8, 9), (6, 9, 10)	77.0694°	QuadAngles
Lattice cell obtuse angle	4, 3, 5), (4, 6, 5), (6, 5, 8), (6, 9, 8)	102.931°	QuadAngles

**Table 2.** Filter specifications for ensemble models in comparison experiment.

Structure Matrix	GCN Filters	Dense Filters

	Single GCN	
$L_{\text{mt}}$	64, 64, 64	256, 32, 8, 1
	2-GCN Ensemble	
$L_{\text{mt}}$	64, 64, 64	256, 32, 8, 1
$L_{\text{mt}}$	32, 32, 32	256, 32, 8, 1
	3-GCN Ensemble	
$L_{\text{mt}}$	64, 64, 64	256, 32, 8, 1
$L_{\text{mt}}$	32, 32, 32	256, 32, 8, 1
$L_{\text{mt}}$	16, 16, 16	256, 32, 8, 1
	2-level GPCN	
*L*_inter_	64, 64, 64	256, 32, 8, 1
$L_{\text{mt}}$	32, 32, 32	256, 32, 8, 1
	3-level GPCN	
*L*_coarse_	64, 64, 64	256, 32, 8, 1
*L*_inter_	32, 32, 32	256, 32, 8, 1
$L_{\text{mt}}$	16, 16, 16	256, 32, 8, 1
	N-GCN (radii 1,2,4)	
$L_{\text{mt}}^{r}$	64, 64, 64	256, 32, 8, 1
	N-GCN (radii 1,2,4,8,16)	
$L_{\text{mt}}^{r}$	64, 64, 64	256, 32, 8, 1

**Table 3.** Mean error and uncertainty of several GCN ensemble models across ten random trials. For each trial, the random seed was set to the same value for each model. Reported values are the minimum error on the validation set during training (not the error at the final epoch). Normalized Mean Squared Error (NMSE) values are unitless. Only one trial was performed with the DiffPool model.

Model Name	Mean NMSE ± Std. Dev (× 10 ^−3^)	Min NMSE (× 10^−3^)

Single GCN	1.55 ± 0.10	1.45914
Ensemble—2 GCNs	1.44 ± 0.07	1.38313
Ensemble—3 GCNs	1.71 ± 0.20	1.43059
2-level GPCN	1.43 ± 0.12	1.24838
2-level A-GPCN	0.17 ± 0.05	0.08963
3-level GPCN	2.09 ± 0.32	1.57199
3-level A-GPCN	0.131 ± 0.030	0.10148
N-GCN radii (1,2,4)	1.30 ± 0.05	1.23875
N-GCN radii (1,2,4,8,16)	1.30 ± 0.06	1.22023
DiffPool	2.041 ± n/a	2.041

**Table 4.** Mean wall-clock time to perform feed-forward and backpropagation for one batch of data, for various GCN ensemble models. Times were collected on a single Intel(R) Xeon(R) CPU core and an NVIDIA TITAN X GPU.

Model Name	Mean time per batch (s)

Single GCN	0.042
Ensemble—2 GCNs	0.047
Ensemble—3 GCNs	0.056
2-level GPCN	0.056
2-level A-GPCN	0.056
3-level GPCN	0.061
3-level A-GPCN	0.059
N-GCN, radii (1, 2, 4)	0.067
N-GCN, radii (1, 2, 4, 8, 16)	0.086
DiffPool	0.0934

## Footnotes

1 Other GCN research uses powers of the Laplacian, the normalized Laplacian, the symmetric normalized Laplacian, etc. Comparison of these structure matrices is beyond the scope of this paper.

2 AssociationandangleconstraintsweresufficienttoreplicatethebendingresistancebehaviorofMTs.Wehopetorunasimilarexperiment using higher-order particle interactions (which may be more biologically plausible), in future work.

3 The LAMMPS manual uses the character K to represent the interaction coefficient; we have used L to distinguish it from the spring constant k, for which we have $L=\frac{k}{2}$.

4 Meaning summed over all monomers, in contrast to the per-monomer predictions made in section 4.
